# Multimodal imaging reveals the spatiotemporal dynamics of recollection

**DOI:** 10.1016/j.neuroimage.2012.11.030

**Published:** 2013-03

**Authors:** Zara M. Bergström, Richard N. Henson, Jason R. Taylor, Jon S. Simons

**Affiliations:** a Department of Psychology, University of Cambridge, Downing Street, Cambridge CB2 3EB, UK; b Behavioural and Clinical Neuroscience Institute, University of Cambridge, Downing Street, Cambridge CB2 3EB, UK; c MRC Cognition and Brain Sciences Unit, 15 Chaucer Road, Cambridge CB2 7EF, UK

**Keywords:** Episodic, Recollection, PFC, Precuneus, Multimodal, MEG, fMRI

## Abstract

Functional MRI research suggests that different frontal and parietal cortical regions support strategic processes that are engaged at different stages of recollection, from pre-retrieval processing of a cue to post-retrieval maintenance and evaluation of recollected information. Whereas some of these regions respond in a domain-general way, other regions are sensitive to the type of information being recollected. However, the low temporal resolution of fMRI cannot distinguish component processes at the time-scale at which recollection occurs. We therefore combined fMRI with the excellent temporal resolution of source localised EEG/MEG to investigate the spatiotemporal neural dynamics of recollection. fMRI and EEG/MEG data were collected from the same participants in two sessions while they retrieved different types of episodic information. This multimodal imaging approach revealed striking consistency between the regions identified with fMRI and EEG/MEG, providing novel evidence of how these brain areas interact over time to support source recollection. For domain-general recollection, results from both modalities converged in showing the strongest activations in medial parietal cortex, which according to EEG/MEG was reliable at a late retrieval stage. Domain-specific source recollection increased fMRI and EEG/MEG activation in the left lateral prefrontal cortex, which EEG/MEG indicated also to be recruited during a post-recollection stage. The findings suggest that although medial parietal and left lateral prefrontal regions mediate functionally different retrieval processes, they are both engaged at a late stage of episodic retrieval.

## Highlights

► Frontal and parietal cortical regions activate during conscious recollection. ► The timing of involvement of these regions during retrieval is unknown. ► We combined fMRI with the excellent temporal resolution of source-localised EEG/MEG. ► Imaging modalities showed striking spatial convergence in fronto-parietal areas. ► The EEG/MEG time-course indicated a post-retrieval role for convergence regions.

## Introduction

Intentional recollection of past experiences involves a series of successive stages, from initial targeted analysis of a retrieval cue that biases retrieval towards a particular type of memory information, to monitoring the retrieved information for diagnostic qualitative characteristics and evaluating these against response criteria (Fletcher and Henson, 2001). Whereas the hippocampus is critical for matching information in a cue with a stored episodic trace, pre- and post-retrieval processes are thought to be mediated by a network of cortical regions that interact with the hippocampus during recollection (Moscovitch, 1992; Simons and Spiers, 2003). Previous fMRI research has consistently shown enhanced activation in posterior parietal (PPC) and left lateral prefrontal (LPFC) cortical regions during tasks that require retrieval and monitoring of contextual information, such as source memory judgements (Johnson et al., 1993), compared to simple item recognition tasks (Dobbins et al., 2002; Mitchell and Johnson, 2009). These findings suggests that PPC and left LPFC regions are particularly recruited to facilitate intentional recollection and are less involved when behaviour is based on more automatic forms of memory. PPC activation is often domain-general, that is, independent of the type of information retrieved (Duarte et al., 2011; Hornberger et al., 2006). In contrast, left LPFC activity is often enhanced when people are asked to recollect conceptual/verbal compared to perceptual/non-verbal details of an event, indicating a domain-specific role in recollection (Dobbins and Wagner, 2005; Simons, Owen et al., 2005).

Different hypotheses associate PPC and left LPFC with either pre- or post-retrieval stages. For example, one hypothesis suggests that both medial and lateral parts of the dorsal PPC are involved in top-down attention to memory during pre-retrieval search (Cabeza et al., 2008). In contrast, other research has indicated that PPC activation may be related to metacognitive reflection on the quality of retrieved memories (Chua et al., 2006), which may involve elaborative processing of already recollected information (Daselaar et al., 2008). Left LPFC has been suggested to support the conceptual processing of retrieval cues at a pre-retrieval stage of recollection in order to bias the retrieval search process towards conceptual information in memory (Cabeza et al., 2003). An alternative view suggests that left LPFC activations reflect systematic monitoring of highly differentiated information during memory judgements (Nolde et al., 1998). Each of these accounts makes a prediction about the relative time-courses of PPC and left LPFC during retrieval; however, interpreting temporal information from fMRI data is problematic because the haemodynamic response effectively integrates several seconds of neural activity, whereas each stage of recollection likely unfolds over fractions of a second. Thus, previous attributions of left LPFC and PPC to particular stages of retrieval have tended to be based on indirect task manipulations rather than direct evidence of neural activation timing.

Event-related potentials (ERPs) measure neural activity at a millisecond scale, and have revealed retrieval-related neural effects that have been tentatively attributed to LPFC and PPC based on functional profiles and topographic distributions over sensors. Because of their temporal characteristics, some of these effects are interpreted as correlates of early versus late retrieval stages (Johansson and Mecklinger, 2003; Rugg and Wilding, 2000). The earliest ERP signs of episodic recollection emerge from around 450 ms after stimulus presentation in the form of an enhanced parietal positive peak, typically left lateralised, referred to as the “parietal old/new effect” because it is often observed during recognition memory tasks when comparing correctly recognised “old” items with correctly rejected “new” items (Rugg and Curran, 2007). This effect shows similar functional characteristics to fMRI activations in the left inferior lateral PPC, and has therefore been hypothesised to generate from this region (Vilberg and Rugg, 2008).

Following the parietal old/new effect, intentional recollection is also often associated with enhanced negative slow-drifts over posterior electrodes (e.g. Friedman et al., 2005; Mecklinger et al., 2007; Senkfor and Van Petten, 1998; Wilding, 1999) that have been suggested to reflect processes that are engaged when task-relevant memory features are not readily recovered or need continued evaluation (Johansson and Mecklinger, 2003; see also Herron, 2007). The late posterior negativity (LPN) is enhanced when participants report that they vividly remember an episode as opposed to have a feeling of familiarity (Leynes and Phillips, 2008), and when participants make metamemory judgements rather than old/new recognition decisions (Wolk et al., 2007), similar to the PPC fMRI activations described above (Chua et al., 2006). The parietal distribution and functional profile of the LPN has led researchers to suggest that it may also originate in domain-general PPC (e.g. Johansson and Mecklinger, 2003). In contrast, domain-specific ERP effects are typically seen over frontal electrode sites relatively late after stimulus presentation. Similarly to frontal fMRI activations, late frontal ERP slow-drifts have been found to distinguish between intentional retrieval of conceptual and perceptual contextual information (Mecklinger et al., 2007; Wilding, 1999).

One might be tempted to assume that frontal and parietal ERP effects originate from directly underlying cortical regions and thus are generated by the same frontal and parietal regions that show recollection-related fMRI activations. However, EEG scalp distributions cannot be easily interpreted because it is impossible to determine uniquely the underlying neural generators of scalp-recorded electrophysiological effects (the “inverse problem”; Nunez and Srinivasan, 2006). Furthermore, EEG fields are distorted by passing through skull and scalp tissue, and the resulting scalp distributions are highly sensitive to choice of reference site, so the maximum site of an EEG effect will differ depending on this arbitrary choice. Finally, on a more fundamental level, the relationship between neural firing as measured by electrophysiology and the haemodynamic fMRI signal is not yet fully understood (Logothetis, 2008). Since these techniques are measuring complementary aspects of neural activity, there are many possible situations where effects in one modality may be invisible in the other (e.g. Ekstrom, 2010). Links between imaging modalities have therefore been highly speculative, with attributions of EEG/MEG sensor effects to specific brain regions based on uncertain evidence.

Methods for mathematically estimating the underlying cortical generators of scalp-level EEG effects have become increasingly sophisticated over recent years. It is advantageous to combine EEG with magnetoencephalography (MEG) recordings in these studies (Sharon et al., 2007), since the latter have the additional benefit of being reference-free and are not distorted by passing through tissue. Solving the inverse problem is non-trivial, but constraining the localisation of activation to participants' cortical sheet as estimated by their individual structural MRI (Mattout et al., 2007), coupled with various other methodological advances such as fusing of EEG and MEG information during the inverse reconstruction (Henson et al., 2009) has produced highly promising results. For early perceptual processes such as object recognition, results with these novel source localisation techniques show high spatial overlap with fMRI activations (e.g. Bar et al., 2006). Because EEG/MEG localisation techniques require untestable starting assumptions to solve the inverse problem, inevitably affecting the outcome (Pascual-Marqui, 1999), demonstrating spatial convergence between independent imaging modalities is particularly strong evidence for isolating the neural correlates of a task or cognitive process. No previous study has demonstrated converging fMRI and EEG/MEG source localisation of domain-general and domain-specific strategic recollection processes, as investigated here.

We collected fMRI and EEG/MEG data from the same group of participants in two separate sessions while they undertook an intentional recollection task where they had to remember different types of source information about a previously presented item. During non-scanned study phases, participants viewed pictures of famous faces presented either on the left or the right of the screen, and made either pleasantness or semantic judgements about each face. fMRI and EEG/MEG data was collected during subsequent test phases while participants were shown the faces again and asked to remember either the location where the face picture had been presented (focusing retrieval towards visuospatial memory information), or which task they had undertaken on the picture (focusing retrieval towards conceptual memory information). In a control condition, participants made semantic judgements about pictures of famous faces that were novel in the experimental context. Within each imaging modality, we looked for common activation during both types of recollection when contrasted against the control condition in order to investigate domain-general retrieval processes. Brain activity associated with the different recollection tasks was contrasted in order to investigate the neural basis of domain-specific retrieval processes.

We then estimated the cortical generators of scalp level EEG/MEG effects, seeking convergence with fMRI to characterize the spatiotemporal dynamics of recollection. The fMRI data were predicted to show domain-general activation in PPC and domain-specific activation in left LPFC. Regions associated with “early” pre-retrieval stages should show EEG/MEG effects during the first few hundred milliseconds after cue presentation, before the first ERP signs of conscious recollection (i.e. the parietal “old/new” effect, Rugg and Curran, 2007). Effects in regions mediating “late” post-retrieval processing should emerge after the first ERP signs of conscious recollection have occurred.

## Material and methods

### Participants

Eighteen right handed participants (8 males, mean age 25, age range 19–35) completed the fMRI and EEG/MEG versions of the experiment in two separate sessions, at a minimum of 7 days apart. Half the participants completed the fMRI session first and the other half completed the EEG/MEG session first. Task design was identical across the sessions but each used a different set of stimuli (counterbalanced across participants). Written informed consent was obtained from participants in a manner approved by the University of Cambridge Psychology Research Ethics Committee.

### Stimuli

Four-hundred and thirty-two black and white photographs of famous faces (216 actors of which 108 were British nationals and 108 were not British, and 216 non-actors of which 108 were British and 108 were not British) were used as experimental stimuli. We used famous faces as stimuli to maximise recollection accuracy, in line with previous research (e.g. Simons, Owen et al., 2005). These photographs were divided into six lists of 72 items in each, containing equal proportions of actors and British nationals that were also matched on gender distribution. Three of the lists were always used during the first imaging session and the other three during the second imaging session in order to counterbalance stimuli across imaging modality. For each participant, two of the lists were presented during study and subsequently tested with instructions to recollect visuospatial or conceptual information, whereas one list was assigned during the test phase to the control condition. List assignment to study and retrieval test conditions was fully counterbalanced across participants.

### Tasks and procedure

Upon arrival, participants completed a full cycle of study and test practice. Subsequently, they were brought into the MRI scanner or EEG/MEG machine, and undertook six study-test cycles (where study phases were ~ 2.5 min and test phases ~ 6.8 min) with short breaks in between each block. Only the test phases were scanned.

**Fig. 1:**
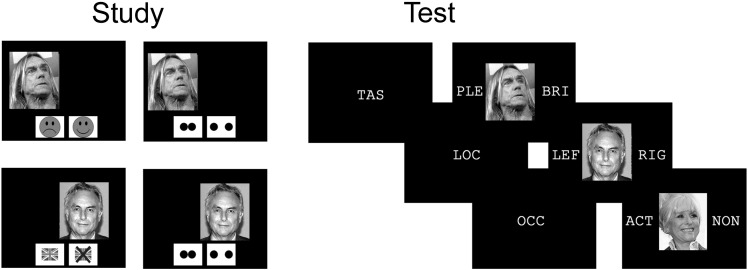
Stimuli examples and procedure overview. During study phases, pictures of famous faces were presented on the left or right of the screen, either slightly nearer or further out from the centre. Participants were first pseudo-randomly cued to make either a pleasant/unpleasant or a British/non-British judgement about each face. Second, they made a near/far perceptual judgement on the picture location. During the test phases, participants were pseudo-randomly cued to make context memory or semantic control decisions about stimuli. In context memory conditions, decisions required recollection of either whether the pleasant/unpleasant or British/non-British task had been undertaken (Task recollection) on whether that stimuli had been shown on the left or right side of the screen (Location recollection). In the Semantic control condition, participants made actor/non-actor occupation decisions about experimentally novel famous faces.

During the study phases, participants were shown pictures of famous faces presented on the left or the right of the screen, at two different distances from the screen centre (along the horizontal axis), either slightly nearer or further away from the middle. They were asked to perform two different judgements on each face, as indicated by prompt symbols at the bottom of the screen. The first question differed from trial to trial, and involved either making a Britishness judgement or a pleasantness judgement about the famous person/face depending on which prompt was shown (see Fig. 1 for stimuli examples). On each trial, after their first judgement but while the face picture was still on the screen, the prompt changed to indicate that participants should judge whether the picture was presented near or far away from the middle. The purpose of this second task was to ensure that participants made an interactive judgement based on the location of the stimulus, so that both subsequent task and location recollection would involve recollection of an interactive as opposed to independent context feature. That is, an interactive context feature is one that influences the way a particular item is processed and encoded, whereas an independent context feature does not (see Baddeley, 1982), and these encoding differences could potentially influence subsequent recollection. We therefore aimed to control for differences in interactive/independent context between Task and Location conditions, in contrast to previous research that has typically overlooked this issue. However, it should be noted that whereas remembering which pleasantness or Britishness judgement one made during study was diagnostic for answering the task recollection question, remembering whether one made a near or far judgement during study was not diagnostic for answering the location recollection question.

The order of task and stimulus location across trials was pseudo-randomised using Mix software (Van Casteren and Davis, 2006) with no more than three repetitions of the same experimental condition. Each study trial began with either the pleasantness or Britishness prompt displayed for 1000 ms, which was followed by the face picture together with the prompt for 2400 ms, a 100 ms black screen, the face and distance prompt for 2400 ms, and another 100 ms black screen.

During the test phases, participants were presented with the same pictures of famous faces seen during the preceding study phase intermixed with pictures of new famous faces. Participants were again asked to make different types of judgements depending on prompt symbols presented prior to each face picture, in the centre of the screen (Fig. 1). If the prompt consisted of the three letters ‘LOC’ (for location), participants should try to remember whether the upcoming picture had been presented on the left or the right of the screen during the previous study phase (Location recollection). If the prompt consisted of the three letters ‘TAS’ (for task), participants should try to remember which of the two initial judgement tasks they had completed on upcoming picture during the previous study phase, the Britishness or Pleasantness judgement (Task recollection). The aims of these two tasks were to bias recollection towards visuospatial/non-verbal details during Location recollection and towards conceptual/verbal details during Task recollection. Therefore, our design manipulated the type of information participants should focus on during retrieval (similar to the concept of “retrieval orientation”, Rugg and Wilding, 2000) while keeping the actual context encoding constant (cf. Dobbins and Wagner, 2005, for a similar operationalisation). Finally, if the prompt consisted of the three letters ‘OCC’ (for occupation), participants were shown a new famous face they had not seen in the previous experimental context, and made a judgement regarding whether or not the famous person was an actor by occupation^1^ (Semantic control). This task was included as a non-episodic control condition for investigating brain activity commonly elicited by both recollection conditions whilst controlling for perceptual, decision, and motor demands, and was closely based on previous studies that included a similar baseline task (e.g. Simons, Gilbert et al., 2005; Simons, Owen et al., 2005; Simons et al., 2008. Thus, our task design was aimed towards distinguishing strategic episodic processes from other types of generic decision processes, such as those associated with semantic retrieval, but should not be directly compared to an old/new recognition task since the control condition differed from the episodic conditions both in terms of old/new status and task instructions.

Test trials were presented in a pseudorandom order with no more than two repetitions of the same experimental condition. Each trial began with a fixation cross with a duration jittered between 500 and 1500 ms, after which the prompts were presented, with a duration jittered in 1000 ms steps between 2000 and 12 000 ms, drawn from an exponential distribution with a mean duration of 4600 ms, to achieve a higher effective sampling rate over trials. Finally, the face picture was presented in the centre of the screen for 3000 ms. For all judgements, participants gave their answers by pressing buttons using the index and middle finger of one hand, with response hand counterbalanced across participants.

### fMRI scanning and preprocessing

Echo-planar functional images (TR = 2.25 s; TE = 30 ms; 36 sequentially acquired axial slices oriented ~ 10–20° to the AC–PC transverse plane; 2 mm thick, with a 3 × 3 mm in-plane resolution and 1 mm gap; 64 × 64 pixels; 78° flip angle) and high resolution, T1-weighted structural MPRAGE images were collected using a 3T Siemens TIM Trio System. EPI data was collected as a separate functional session for each of the six retrieval tests with 195 volumes in each, and the first six volumes in each run were discarded to allow for T1 equilibration.

Images were pre-processed and analysed statistically using SPM 8 (Wellcome Department of Imaging Neuroscience, London). Functional images were first spatially realigned to the first image to correct for movement then corrected for differences in slice timing by resampling all slices with respect to the middle slice. Structural scans were coregistered to the mean realigned functional image for each participant and segmented into grey and white matter and cerebro-spinal fluid, and the grey matter was normalised to the grey matter in an average T1 template in Montreal Neurological Institute (MNI) stereotactic space (Cocosco et al., 1997). The structural normalization parameters from the segmentation step were used to spatially normalize the realigned functional images, which were subsequently smoothed with an 8 mm FWHM Gaussian kernel.

### fMRI statistical analysis

A mixed effects statistical analysis was undertaken in two stages. First, a subject-specific fixed effects model was created by convolving a boxcar function corresponding to stimulus duration (3 s) beginning at the onset of each event of interest with a canonical hemodynamic response function. The time series at each voxel, and the regressors in the model, were high-pass filtered with a 1/128 Hz cut-off to remove low frequency noise, and an AR(1) model was used to correct for temporal autocorrelation in the residual error. Face picture onsets for Location recollection, Task recollection and Semantic control trials were modelled separately, including only trials with correct responses. Three additional regressors modelled cue onsets for the three conditions, and one regressor modelled errors and missed responses. Parameters for each regressor were estimated including session specific effects and movement parameters as confound covariates. Regressors from the different sessions were averaged together into separate contrast images for Task, Location and Semantic control items, and the resulting contrast estimates were entered into a group level factorial GLM. SPM T-images were estimated for each paired condition comparison, treating subjects as a random effect. fMRI activations were considered significant if they exceeded a threshold of uncorrected p < 0.001, with a minimum extent of 10 voxels (a threshold that should provide an adequate balance between Type I and II errors; Lieberman and Cunningham, 2009). Domain-general recollection effects were investigated by an inclusively masked analysis that assessed which regions showed significant differences between both Task and Location recollection compared to Semantic control trials. A conjunction analysis was used in order to identify the peak voxels within the common activations where the overlap between the two effects was strongest.^2^ Domain-specific recollection effects were assessed by directly contrasting Task and Location recollection trials. The approximate Brodmann's areas of significant clusters were estimated using the Talairach and Tournoux (1988) atlas and the Talairach daemon software, after adjusting coordinates to allow for differences between the MNI and Talairach templates (http://imaging.mrc-cbu.cam.ac.uk/imaging/MniTalairach).

### EEG/MEG recording and preprocessing

EEG and MEG data were recorded simultaneously in a light Elekta-Neuromag magnetically-shielded room using an Elekta Neuromag Vectorview machine with 102 magnetometers, 204 planar gradiometers and 70 scalp EEG channels. EEG electrodes were positioned in an Easycap EEG cap with standard electrode locations from the extended 10/20 system referenced to a nose recording, including additional bipolar channels measuring vertical and horizontal EOG. Participant head position in relation to MEG sensors was monitored continuously with Head-Position Indicator (HPI) coils that were attached to the outside of the EEG cap and emitted high-frequency sinusoidal currents (293–321 Hz). The location of HPI coils, EEG channels and general head shape was measured relative to three anatomical fiducials (the nasion and left and right pre-auricular points) using a 3D digitizer (Fastrak Polhemus Inc., Colchester, VA). Data were sampled at 1 kHz with a band-pass filter from 0.03–330 Hz.

All data were first downsampled by a factor of 4 (which included applying an anti-aliasing filter) to reduce processing time and remove high frequency noise, using the MaxFilter 2.0 software (Elekta-Neuromag) MEG magnetometer and gradiometer data were cleaned of external magnetic noise using the temporal extension of Signal-Space Separation (Taulu et al., 2005) with a 10 s window and 0.9 correlation threshold, as implemented in MaxFilter. Bad MEG channels were identified either manually or using MaxFilter's automatic function and recreated by MaxFilter, and head movement was corrected with a 200 ms interval.

Next, data were imported into SPM 8 and electrode positions were recalculated based on digitized positions. EEG, MEG magnetometer and MEG gradiometer data were separately reduced to 65 orthogonal components for each modality using Principal Component Analysis and submitted to extended infomax Independent Component Analysis (ICA) using runica from the EEGLAB toolbox with default extended-mode training parameters (Delorme and Makeig, 2004). Independent components reflecting eye movements were identified by their correlation with VEOG and HEOG raw time-series, and ECG and EMG noise components were identified by visual inspection of component scalp topographies and time courses. Noise components were discarded from the data by back-projecting all but these components to the data space.

Subsequently, all remaining data were further low-pass filtered with a 40 Hz cut-off (a two-pass 5th-order Butterworth digital filter). This second step of filtering was done to remove residual high frequency noise not captured by the ICA (we did not filter before ICA because the analysis may use high frequency information to extract components). EEG and MEG data were epoched from − 500 ms to 2500 ms post-stimulus, time-locked to the onset of the face stimuli during the memory test phases (removing the mean baseline from − 100 ms to 0 ms). Any epochs that contained flat-lined EEG or MEG or high amplitude artefacts were rejected (amplitude thresholds were 500 fT for magnetometers, 2000 fT/m for gradiometers and 150 μV for EEG, on average 8% of trials were deleted in total). Event-Related Potentials (ERPs) and Event-Related Fields (ERFs) were created by averaging the remaining epochs separately for the Task recollection, Location recollection and Semantic control conditions, including only accurate trials (mean trial number per condition: 51 [range 36–64, which is equivalent to 50–89% of trials in this condition], 56 [range 29–72, 40–100%] and 51 [range 35–64, 49-89%] respectively).

Since ERPs and ERFs primarily capture low frequency effects that are time-locked to stimulus onset, an additional analysis tested whether the experimental conditions were associated with significant differences in both induced and evoked time-frequency power changes by analysing single trials with wavelet decomposition between 4 and 40 Hz and averaging the resulting power estimates for each condition. However, this analysis did not reveal any convergent findings between fMRI and EEG/MEG localisation and is therefore not discussed further.^3^

### EEG/MEG sensor-level statistical analysis

A first analysis assessed significant ERP/ERF modulations at the sensor level in order to relate those to source localised EEG/MEG effects in the following step. The purpose of this analysis was to enable interpretation of source-level effects with regards to well-known ERP components, and to validate that significant findings in source space only emerged in time-windows when scalp-level effects were reliable. For each participant, each time sample of their ERPs and magnetometer ERFs was projected onto a 2-dimensional 64 × 64 voxel sensor topography, separate for each condition and modality. These 2D planes were concatenated across time to create a 3-dimensional topography-by-time volume, which was smoothed with a 6 mm × 6 mm × 6 ms Gaussian filter. Resulting 3D images were entered into a group level factorial GLM, and SPM F-images were estimated for each paired condition comparison. Domain-general recollection effects were investigated by an inclusively masked analysis that assessed which portions of the spatiotemporal volume showed significant differences between both Task and Location recollection compared to Semantic control trials. Domain-specific recollection effects were assessed by directly contrasting Task and Location recollection trials. Effects were accepted as significant if they exceeded a corrected cluster threshold of P < 0.05, and an uncorrected height threshold of P < 0.001. The sensor level gradiometer ERFs were not analysed statistically due to difficulties associated with applying Random Field Theory and parametric tests to gradiometer scalp data (see http://imaging.mrc-cbu.cam.ac.uk/meg/SensorSpm), but gradiometer ERFs were included in the source localisation (next section).

### EEG/MEG source localisation

The inverse of the transformation for normalising each participant's structural MRI grey matter to the MNI template brain (see fMRI analysis section) was used to warp a cortical mesh from a template brain in MNI space to each participant's MRI space (Mattout et al., 2007), creating an individualized mesh with 8196 vertices (“normal” resolution). EEG and MEG data were projected onto each participants MRI space by a rigid-body coregistration based on minimising the sum of squared differences between the digitised head points (and electrode positions for EEG) and this scalp mesh. A forward model was created by fitting a single sphere for MEG and a Boundary Element model (BEM) for EEG to the scalp mesh and computing normally oriented lead-fields for a dipole at each point in the cortical mesh.

In order to estimate the distributed cortical sources of sensor-level ERP and ERF data, the lead-field matrix was subsequently inverse reconstructed. EEG, MEG gradiometer and MEG magnetometer data were fused using a Parametric Empirical Bayesian approach that has been demonstrated to improve source solutions compared to unimodal inversions (Henson et al., 2009). The inversion was conducted with a minimum norm criterion, including the whole epoch. A minimum norm criterion was chosen since it involves minimal assumptions beyond favouring the source solution that entails minimal total energy, and has therefore been recommended when studying higher cognitive functions where the generators are unknown (see Hauk, 2004). Source estimates were summarised as contrast images across successive 200 ms windows between 0 and 2400 ms, including all frequencies in the ERP/ERF average, and smoothed with an 8 mm FWHM Gaussian kernel. These images were then entered into a group level factorial GLM, and SPM T-images were estimated for each paired condition comparison, with the statistics restricted to voxels within a grey-matter mask image. In line with the fMRI and sensor-level analyses, an inclusive masking analysis assessed which cortical regions showed significant energy differences between both Task and Location recollection compared to Semantic control trials. Domain-specific recollection effects were assessed by directly contrasting Task and Location recollection trials. Effects were accepted as significant if they exceeded a threshold of uncorrected P < 0.001, with a minimum extent of 10 voxels. Importantly, this analysis thus anatomically constrained EEG/MEG localisation only based on structural and not functional MRI information, meaning that there were no methodological reasons why the functional imaging modalities would show spatial convergence. Rather, any such convergence could be confidently interpreted as due to each modality *independently* identifying a region as showing significant activity modulations based on the experimental manipulations.

### Statistical convergence between fMRI and EEG/MEG source localisation

The final concluding analysis directly compared the overlap between independently analysed EEG/MEG source activation and fMRI data by inclusively masking the EEG/MEG source localisation for domain-general and domain-specific effects in each successive 200 ms window (described above) with the corresponding functional contrast in the fMRI analysis. As both modalities had an individual height threshold of P < 0.001, the joint probability for convergent activations in this analysis was in the order of P < 1.5 × 10^− 5^ (Fisher, 1990), although since the domain-general analysis already involved an inclusively masked analysis for two pairwise contrasts in each modality, the statistical threshold for this analysis was actually even more conservative.

## Results

### Behavioural results

There were no significant differences in behavioural performance between imaging modalities or sessions in neither study nor test phases, so all behavioural data are presented collapsed across session and modality.

In the study phases, participants were on average more likely to respond that faces were pleasant than unpleasant (mean proportion pleasant judgement = 0.67, SEM = 0.03, RT (ms) = 1411, SEM = 41) and accuracy at the Britishness judgement was significantly above chance (mean proportion accurate = 0.77, SEM = 0.02, RT (ms) = 1431, SEM = 39).

Test phase data are presented in Table 1. Accuracy in all conditions was high and significantly above chance, but was slightly higher for the Location recollection judgements compared to the other two conditions (Location recollection vs. Semantic control: t(17) = 2.4, P < 0.05; Location vs. Task recollection: t(17) = 2.8, P < 0.05) whereas there was no difference between Task recollection and Semantic control accuracy (t < 1, n.s.). Both Semantic control (t(17) = 6.8, P < 0.001) and Location recollection (t(17) = 8.9, P < 0.001) judgements were however significantly faster than Task recollection judgements. There were however only trend level faster RTs for Location recollection than Semantic control judgements (t(17) = 1.8, P < 0.1).

In sum, behavioural performance was high in all conditions, and there was no simple relationship between accuracy and reaction times, suggesting that the conditions did not differ systematically in general “difficulty” levels.

## fMRI results

### Domain-general fMRI activations

The inclusive masking analysis revealed a typical network of fronto-parietal regions as more active during both types of recollection than during semantic retrieval trials (Table 2). There was a large cluster of activation across bilateral PPC, including both superior and inferior parts of medial and lateral PPC, with activation peaking in a medial PPC region in the left Precuneus. Additional domain-general recollection-related activation was evident bilaterally in lateral anterior and dorsolateral PFC, bilateral Cingulate Gyrus, bilateral Premotor Cortex and right Insula.

The reverse contrast – testing for regions more active during semantic judgements on experimentally-novel faces than episodic retrieval – revealed enhanced activation in a large bilateral medial PFC cluster encompassing caudal Orbitofrontal and Anterior Cingulate cortices, as well as clusters in bilateral Medial Temporal Lobes, bilateral Posterior Cingulate and a small cluster in the right ventrolateral PFC (Table 2).

### Domain-specific fMRI activations

Regions more active during Task than Location recollection included a large left LPFC cluster encompassing both dorsal and ventral LPFC, as well as regions in the left inferior lateral PPC, bilateral dorsomedial PFC, and smaller clusters in the right dorsolateral PFC, bilateral Posterior Cingulate, right Supramarginal Gyrus, left Precuneus, bilateral Basal Ganglia and left Middle Temporal Gyrus (Table 3). There were only a few smaller clusters that showed enhanced activation for Location compared to Task recollection, including regions in the right Middle Temporal Gyrus, as well as bilateral Postcentral Gyrus, right Insula and right Precuneus (Table 3).

In sum, the fMRI results showed the predicted pattern with a PPC maximum for domain-general recollection effects, and domain-specific effects with enhanced activation for Task compared to Location recollection in the left LPFC.

### Sensor-level EEG/MEG results

**Fig. 2:**
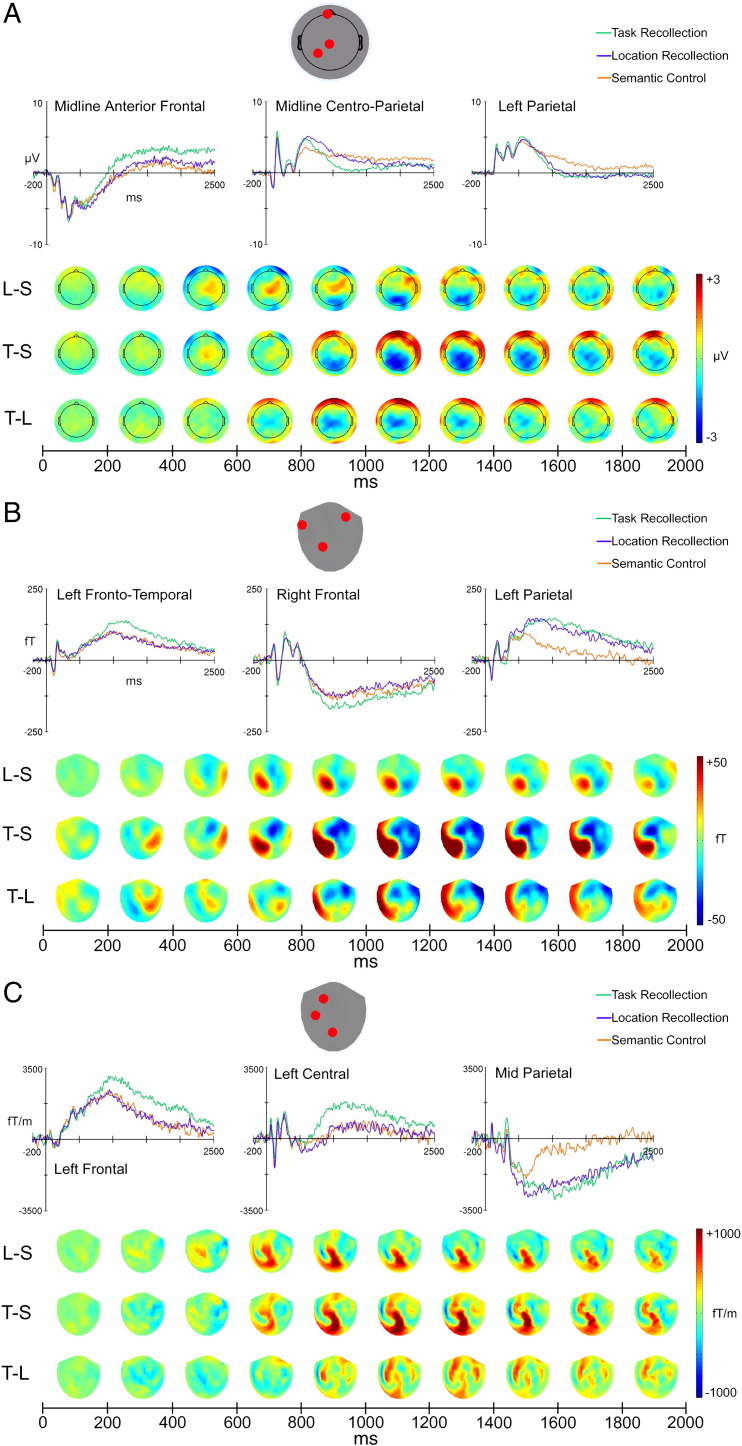
Grand average ERPs (A), and magnetometer (B) and gradiometer (C) ERFs. ERPs/ERFs are plotted for the three conditions at representative sensors where experimental effects were maximal, and topographic maps below depict ERP/ERF amplitude differences between conditions, averaged over successive 200 ms time-windows between 0 and 2 s post-stimulus (Semantic Control subtracted from Location Recollection [L − S], Semantic Control subtracted from Task Recollection [T − S] and Location Recollection subtracted from Task Recollection [T − L]). The gradiometer maps were constructed by computing the root mean square (RMS) of gradiometer pairs for each condition and subtracting these condition RMS maps. All sensor-types showed domain-general late slow-drifts over parietal sensors and more frontally distributed domain-specific late slow-drifts.

The sensor-level EEG and MEG data showed that electrophysiological differences between the three conditions were primarily manifested as relatively late onset, sustained slow-drift effects (Fig. 2). Both types of episodic recollection were associated with an initial centro-parietal positivity in the ERPs around 450–800 ms (see Fig. 2A topographic maps) compared to the Semantic control condition, resembling a typical parietal old/new effect (Rugg and Curran, 2007).^4^ The later part of this parietal positivity was modulated by a large negative slow-drift in EEG (Fig. 2A) and an orthogonally oriented dipolar parietal field in MEG magnetometers (Fig. 2B). MEG gradiometer data (Fig. 2C) indicated that the maximum magnetic field gradient of this late effect was located over the midline posterior sensors. Task recollection was specifically associated with a late frontally distributed slow drift in EEG (Fig. 2A) which had a left fronto-temporal maximum in MEG magnetometers (Fig. 2B) with a maximum magnetic field gradient over left frontal sensors (Fig. 2C).

**Fig. 3:**
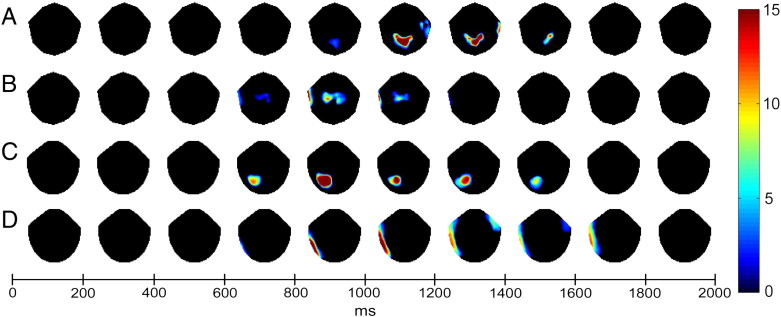
Topographic plots of SPM F-statistics for scalp-level ERPs (A & B) and Magnetometer ERFs (C & D). Maps have been thresholded at P < 0.001, corrected for cluster extent at P < 0.05, and averaged over successive 200 ms time-windows between 0 and 2 s post-stimulus. For domain-general recollection (A & C, testing both types of recollection versus semantic control using inclusive masking), both modalities showed a highly significant parietal effect that was maximal between ~ 1000 and 1400 ms post-stimulus for ERPs (A) and between ~ 600 and 1600 ms post-stimulus for ERFs (C). For domain-specific recollection (B & D, testing the simple difference between Task and Location recollection) both modalities showed a highly significant left temporal/frontal effect that was maximal between ~ 800 and 1200 ms post-stimulus for ERPs (B) and between ~ 800 and 1800 ms for ERFs (D).

Statistical analysis at the sensor level (Fig. 3) confirmed that these late-slow drift effects were highly significant in both ERPs and magnetometer ERFs, whereas the earlier centro-parietal positivity was not significant at the strict corrected threshold (although it was significant at a less conservative height threshold of P < 0.01 uncorrected, which is rather typical for scalp ERP research). The parietal domain-general recollection slow-drift effect was maximal between ~ 1000 and 1600 ms post-stimulus for ERPs and between ~ 600 and 1800 ms post-stimulus for ERFs. The left temporal/frontal effect of domain-specific recollection was maximal between ~ 800–1200 ms post-stimulus for ERPs and ~ 800–1800 ms for ERFs.

## Source-level fused MEEG results

### Domain-general MEEG source activation

**Fig. 4:**
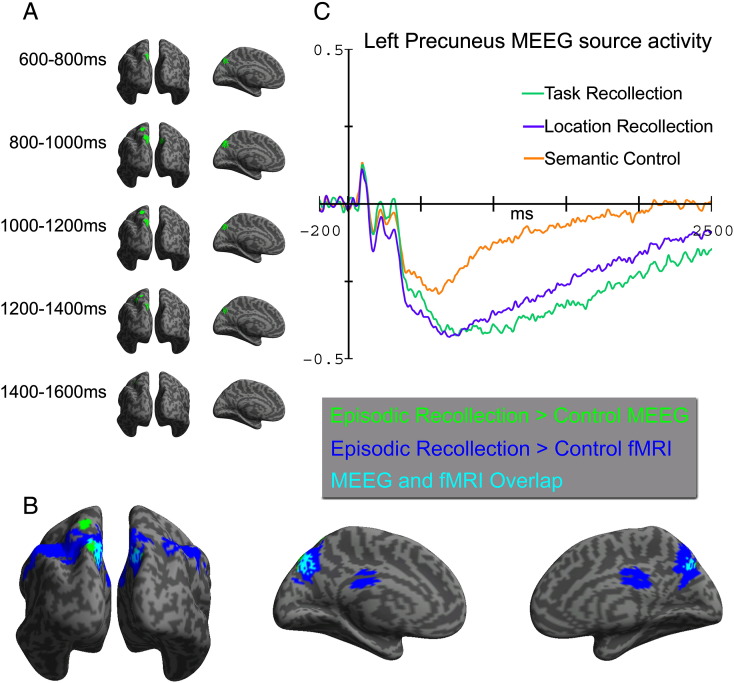
Fused EEG/MEG (MEEG) source localisation results indicating the underlying neural generators of domain-general MEEG recollection effects. Effects are thresholded at P < 0.001 (uncorrected), with a minimum cluster size of 10 voxels. A, an inclusive masking analysis showing regions where both types of episodic recollection produced significantly enhanced MEEG power compared to the Semantic control condition, analysed in successive 200 ms time-windows and overlaid on bilateral posterior (left column) and left medial (right column) inflated canonical cortical surfaces. Domain-general effects were significant at this threshold in a medial and superior PP region between ~ 600 and 1600 ms post-stimulus. B, a direct comparison between the domain-general MEEG localisation in the 800–1000 time-window and the corresponding domain-general fMRI activations overlaid on bilateral posterior (left), left medial (middle) and right medial (right) inflated canonical cortical surfaces, showing substantial spatial overlap in the medial PP cortex. C, estimated MEEG source activity from a 10-voxel sphere centred in the Precuneus region (− 6 − 73 46) that overlapped between MEEG and fMRI modalities (arb. unit).

Inclusive masking of regions showing enhanced MEEG power for both types of episodic recollection compared to semantic retrieval revealed significant activation in the superior medial PPC (Fig. 4A). The peak region in the left Precuneus showed reliable recollection-related differences between approximately 600 and 1600 ms post-stimulus at the P < 0.001 threshold, consistent with the peak timing of the parietally distributed EEG/MEG slow-drift observed at the sensor level. Spatial overlap with fMRI was remarkably consistent (Fig. 4B). The peak voxel that showed the largest fMRI activation for domain-general recollection across the whole head (Table 2) differed by only 5 mm from the corresponding MEEG source peak voxel in the 800–1000 ms time-window when the MEEG effect was maximal (coordinates − 9 − 70 43 and − 6 − 74 42 respectively). Estimated source waveforms from a 10-voxel sphere centred in this region of overlap (Fig. 4C) showed that the source amplitude and temporal profile of the response for both types of recollection was very similar in this region, and that recollection-related differences emerged from around 500 ms post-stimulus. No regions showed significantly enhanced power during semantic control trials compared to episodic recollection.

### Domain-specific MEEG source activation

**Fig. 5:**
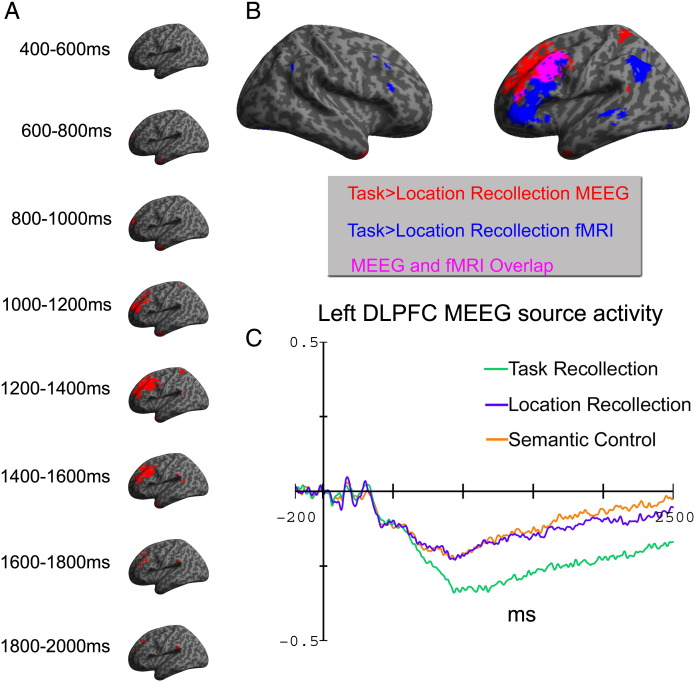
Fused EEG/MEG (MEEG) source localisation results indicating the underlying cortical generators of domain-specific MEEG recollection effects. Effects are thresholded at P < 0.001 (uncorrected), with a minimum cluster size of 10 voxels. A, a simple contrast showing regions where Task recollection produced significantly enhanced MEEG power compared to Location recollection, analysed in successive 200 ms time-windows and overlaid on a left lateral inflated canonical cortical surface. Domain-general effects were maximally significant in left LPFC between ~ 1000 and 1600 ms post-stimulus. B, a direct comparison between the domain-specific MEEG localisation in the 1200–1400 ms time-window and the corresponding domain-specific fMRI activations, showing substantial spatial overlap in the left LPFC cortex. C, estimated MEEG source activity from a 10-voxel sphere centred in the LPFC region (- 42 11 49) that overlapped between MEEG and fMRI modalities (arb. unit).

Directly contrasting regions showing enhanced power during recollection of Task compared to Location context revealed significant activation in the bilateral Temporal pole and left LPFC (Fig. 5A). A small cluster of activation emerged in the left LPFC from around 600 ms onwards, but source activation in this region was particularly enhanced between approximately 1000 and 1600 ms post-stimulus at the P < 0.001 threshold, consistent with the peak timing of the frontally distributed sensor level EEG/MEG slow-drift. Spatial overlap with fMRI was less complete than the domain-general PPC effect, but there was considerable overlap between modalities in the left dorsolateral PFC (Fig. 5B). Estimated source waveforms from a 10-voxel sphere centred in this region of overlap (Fig. 5C) showed that this difference was specifically driven by enhanced power for Task recollection from around 600 ms onwards, whereas the Location recollection and the Semantic control conditions were similar to each other. There were no regions that showed significantly enhanced power during Location recollection compared to Task recollection.

To verify that the lack of significant early differences in PPC and left LPFC were not type II errors due to the relatively strict alpha level of P < 0.001, we also tried lowering the alpha level to a liberal P < 0.01, but there were no significant recollection-related effects in either left LPFC or PPC regions during early time-windows even at this lenient threshold. There was no reliable evidence of significant source activation associated with the early, relatively weaker, centro-parietal old/new ERP positivity in any cortical region.

### Convergence between fMRI and MEEG localisation

**Fig. 6:**
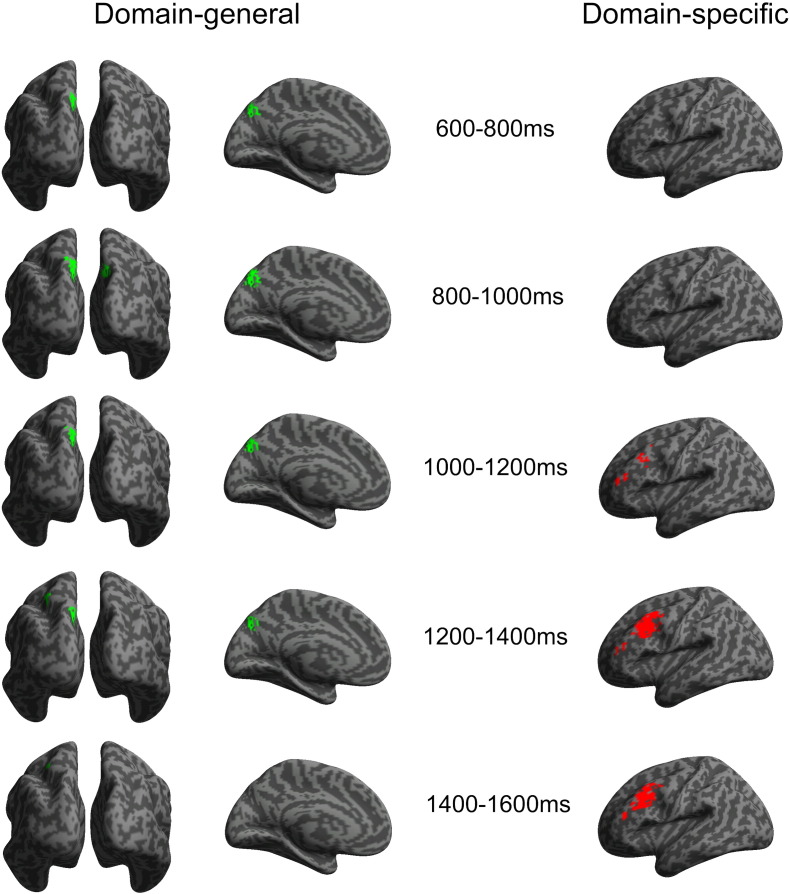
An inclusively masked analysis showing regions that were significantly active in both fMRI and MEEG modalities during domain-general (left) and domain-specific (right) recollection with a joint probability of P < 1.5 × 10^–5^ or less. This analysis confirmed that the Precuneus and left dorsal PFC showed highly significant convergence between fMRI and MEEG for both functional contrasts.

The concluding analysis that directly tested the statistical significance of overlap between our independent imaging modalities confirmed that the observed spatial convergence between fMRI and MEEG effects was extremely unlikely to have occurred by chance (Fig. 6). This inclusive masking analysis, in which convergent activity is considered statistically significant only if it exceeds the conservative threshold of P < 1.5 × 10^− 5^, showed that domain-general recollection effects in the Precuneus were significantly convergent between approx. 600 and 1400 ms. The MEEG effect in this overlapping region between 800 and 1000 ms peaked only 5 mm from the corresponding peak fMRI voxel (see previous section). Overlap between enhanced fMRI and MEEG activation in the left dorsal LPFC during Task recollection was significant between approx. 1200 and 1600 ms post-stimulus, and the MEEG response peaked between 1400 and 1600 ms at a distance of 19 mm from the corresponding peak fMRI voxel (MEEG peak: − 48 18 32; fMRI peak: − 42 11 49, see Table 3).

In sum, source localisation of fused MEEG effects showed a high level of spatial convergence with fMRI activations. Both modalities exhibited maximal domain-general effects in the medial PPC, whereas domain-specific effects were specifically associated with enhanced left LPFC activation. In addition, the MEEG source localisation showed that for both domain-general effects in PPC and domain-specific effects in LPFC, condition differences were manifest as late-onset sustained slow-drifts, with no significant differences observed prior to the 600–800 ms time-window, showing a corresponding time course to the parietally and frontally distributed slow-drifts at the scalp level.

## Discussion

We collected fMRI and EEG/MEG recordings from the same group of participants to investigate the spatiotemporal neural dynamics of source recollection. Using newly developed methods for estimating cortical sources of EEG/MEG (Dale et al., 2000; Henson et al., 2009; Litvak et al., 2011; Mattout et al., 2007), we were able to demonstrate remarkable spatial overlap between independent fMRI and EEG/MEG localisation of domain-general and domain-specific episodic retrieval processes in medial PPC and left LPFC regions respectively. After identifying fronto-parietal regions that showed convergence across modalities, we estimated the EEG/MEG time-course in these regions to investigate the timing of their involvement during stages of recollection. These analyses provide novel evidence that differentially supports the predictions of competing theories of fronto-parietal contributions to recollection, demonstrating the added value of using multimodal imaging as a tool to characterize the brain basis of memory.

Both fMRI and source localised EEG/MEG analyses identified a region in the Precuneus as highly activated during episodic recollection irrespective of the type of information participants were asked to retrieve, consistent with prior fMRI evidence that the PPC supports domain-independent recollection (e.g. Donaldson et al., 2010; Duarte et al., 2011; Hornberger et al., 2006; Wagner et al., 2005). A recent theory of parietal function in memory suggests that both lateral and medial superior PPC regions are involved in the top-down control of episodic retrieval search (Cabeza et al., 2008), predicting that these activations should occur relatively early after presentation of a retrieval cue. Indeed, a recent study found convergent fMRI and source localised MEG activation in the lateral superior PPC with a very early ( < 100 ms) onset of MEG activation when recollection-based judgements were compared to non-mnemonic judgements about previously seen items (Seibert et al., 2011). However, our EEG/MEG data showed no signs of significant activation differences in the medial superior PPC during the early parts of the trial, but rather an enhanced sustained response emerging after around 500 ms post-stimulus. The cause of this discrepancy is unclear since these studies used very dissimilar retrieval tasks, but it may indicate that lateral and medial superior PPC perform different functions during recollection that were differentially recruited across studies, or alternatively, that task differences may have altered the timing of recruitment of a similar neurocognitive process. Although our findings are generally consistent with a strategic role for the medial PPC, the temporal activation profile in this region appears more consistent with a post-retrieval process, such as metacognitive reflection on the quality of memories (Chua et al., 2006; Simons et al., 2010) or post-retrieval elaboration (Daselaar et al., 2008). Alternatively, medial PPC may mediate a retrieval search process that is primarily engaged at a late stage if the retrieval cue fails to automatically bring the goal-relevant contextual features to mind (cf. Johansson and Mecklinger, 2003).

Domain-specific recollection effects showed spatial overlap between fMRI and EEG/MEG modalities in the left dorsal LPFC, where task recollection was associated with enhanced activation compared to both location recollection and semantic retrieval. These results thus corroborate and extend prior fMRI findings suggesting that the left LPFC is engaged during conceptually-based compared to perceptually-based recollection (Dobbins and Wagner, 2005; Simons, Gilbert, et al., 2005; Simons, Owen, et al., 2005). The source localised EEG/MEG time-course showed that this effect was also reliable relatively late after cue presentation, from around 600 ms onwards, and showed a very sustained time course, similar to the PPC response. This result is more consistent with a role for left LPFC in systematic evaluation processes (Nolde et al., 1998) than in semantically guided information production processes (Cabeza et al., 2003) during recollection, as production processes would be expected to occur early after cue presentation, prior to initial signs of recollection. The relatively dorsal location of domain-specific fMRI and EEG/MEG activity in the present data supports the notion of functional specialization within left LPFC, whereby dorsal regions evaluate or manipulate retrieved conceptual information, whilst cue-elaboration processes are mediated by ventral and anterior left LPFC regions (e.g. Daselaar et al., 2008; Dobbins and Han, 2006; Dobbins et al., 2002; Fletcher and Henson, 2001; Simons, Gilbert et al., 2005; Simons and Spiers, 2003).

The current findings are the first strong evidence of the underlying neural generators of widely studied recollection-related ERP effects. The Late Posterior Negativity (LPN) that showed a domain-general response in our experiment has been observed in many prior ERP studies of episodic recollection (e.g. Friedman et al., 2005; Leynes and Phillips, 2008; Mecklinger et al., 2007; Wilding, 1999), and has been associated with strategic processing at a late recollection stage (Herron, 2007; Johansson and Mecklinger, 2003; Wolk et al., 2007), consistent with a generator in PPC regions, as tentatively suggested by some researchers (Mecklinger et al., 2007). Frontally distributed domain-specific ERP slow-drifts have also been described in several previous studies and have been interpreted as indicative of PFC-mediated monitoring processes (e.g. Leynes, 2002; Mecklinger et al., 2007; Wilding, 1999). Since interpreting scalp-distributions of ERP effects in terms of underlying neural generators is notoriously difficult (Nunez and Srinivasan, 2006), our results are the first convincing evidence that recollection-related frontal ERP slow-drifts and the late posterior ERP negativity are generated by left lateral PFC and medial PPC respectively.

The data also contribute novel evidence regarding the sensor-level MEG correlates of domain-general and domain-specific recollection, and how these relate to scalp-effects in the EEG domain. The few prior studies that have investigated MEG correlates of episodic recollection (e.g. Düzel et al., 2003; Evans and Wilding, 2012; Staresina et al., 2005) focused on relatively early MEG effects in recognition tasks, and not on the strategic retrieval processes studied here. By combining EEG with MEG, our research is the first to show that well-known parietal and frontal ERP slow-drifts during recollection are accompanied by corresponding parietal and frontal effects in ERFs.

In addition to demonstrating the usefulness of multimodal imaging for studying the brain basis of retrieval processes, our findings also advance the more general methodological aim of relating fMRI to EEG/MEG measures of neural activity. There is still a great deal of uncertainty regarding the precise relationship between the haemodynamic response and neural activity (Ekstrom, 2010; Logothetis, 2008). It has recently been suggested that both sustained electrophysiological slow-drifts and the fMRI haemodynamic response are related to the excitability level of cortical areas, as indicated by correlations between these imaging measures both during rest and specific tasks (He and Raichle, 2009; Khader et al., 2008; Schicke et al., 2006). This correspondence had previously been largely overlooked in prior multimodal literature, where research has focused largely on convergence between fMRI and source localisation of early, transient ERP/ERF components, such as those associated with perceptually-related memory processes (Bar et al., 2006; Dale et al., 2000; Gonsalves et al., 2005; McDonald et al., 2010). The current experiment has produced novel evidence on this neglected issue by demonstrating that slow cortical potentials and fMRI BOLD are functionally and spatially correlated during conscious recollection (cf. Khader et al., 2007).

Our experiment thus demonstrates that although medial PPC and left LPFC regions are functionally dissociable, they both exhibit a late, sustained response that emerged after initial signs of item-elicited recollection had already occurred (as indicated by an earlier parietal old/new effect in the ERPs; reviewed in Rugg and Curran, 2007). However, several open questions remain regarding the specific roles of these regions. For example, the enhanced response in the left dorsal LPFC during recollection of a previous task indicates that this region may be specifically involved in processing of conceptual or verbal information during episodic retrieval (e.g. Dobbins and Wagner, 2005), but task recollection may also require systematic evaluation of a particularly large number of memory features, which might engage frontally-mediated evaluation processes to a larger extent than other types of memory judgements (Mitchell and Johnson, 2009; Nolde et al., 1998). It is also unclear to what extent LPFC and medial PPC may mediate processes that represent or maintain retrieved information in service of task goals, versus processes that evaluate or manipulate such information. Despite these open issues, our findings constrain future research on left LPFC and medial PPC involvement in episodic recollection towards focusing on a role for these regions at a late retrieval stage.

There are a number of limitations in the inferences that can be drawn from the present data. First, our experimental manipulations primarily affected late ERP/ERF components, and there were no significant sensor-level differences prior to ~ 500 ms after stimulus onset. The source localisation results showed a corresponding pattern, so that source-level effects were only significant when sensor differences were also reliable, demonstrating that the source localisation findings were not spurious but directly related to sensor effects. However, although we have interpreted the absence of early and presence of late EEG/MEG effects in the PPC and left LPFC as evidence that these regions are active at a late retrieval stage, the latency of EEG/MEG divergence between conditions is of course only an upper bound on the time when neurocognitive processing begins to differ. Therefore, it is possible that late EEG/MEG differences between conditions were after-effects produced by earlier neural events that were undetectable by our methods. Evidence against this view comes from the ERP field, where the LPN and late frontal slow-drift effects have been associated with late retrieval processes because of their functional characteristics (discussed in e.g. Johansson and Mecklinger, 2003; Cruse and Wilding, 2009). Nevertheless, although our findings are consistent with a late retrieval role for the PPC and left LPFC, this cannot be conclusively determined from the data.

Second, although we observed highly significant multimodal convergence in PPC and left LPFC, several other regions did not show spatial overlap between modalities. For example, domain-specific recollection effects in the temporal poles were only observed in EEG/MEG, whereas domain-general recollection effects in inferior lateral PPC were specific to fMRI. This discrepancy is in line with previous observations that electrophysiological and haemodynamic measures sometimes dissociate (e.g. Meltzer et al., 2009). However, there are many reasons why these signals may diverge, both in terms of biological factors (e.g. contributions to BOLD changes other than the synchrony of neuronal firing, vascular differences between brain regions, configurations of neurons that do not generate sufficient local-field potentials measurable by EEG/MEG etc., discussed in Ekstrom, 2010) and methodological limitations (e.g. EEG/MEG needs to be filtered to improve SNR but this excludes very high and low frequency oscillations that may contribute to BOLD amplitudes, e.g. Logothetis et al., 2001. Furthermore, MEG is insensitive to radially oriented sources, while fMRI is susceptible to artefacts in some regions such as the temporal poles, etc.). Finally, the minimum-energy assumption in the minimum-norm inversion of the MEG + EEG data (and its degree of regularisation) may have attenuated source estimates in some of the BOLD regions. The multitude of reasons for divergence makes it difficult to speculate on why we observed modality differences in our data, and we therefore limit our inferences to regions that did show independent convergence across modalities.

In conclusion, we used recent advanced multimodal imaging techniques to investigate spatial convergence between independent, complementary neuroimaging measures of recollection-related brain activity. The findings revealed highly significant overlap in medial PPC and left LPFC regions, consistent with the view that these brain areas are reliably involved in facilitating episodic recollection. Using the excellent temporal resolution of source-localised EEG/MEG, we were able to establish the timing of activation effects in these regions with greater precision than previously possible. The results suggest a role for both medial PPC and left LPFC at a late retrieval stage, providing a novel contribution towards the goal of characterizing the brain regions that mediate specific strategic processing stages of recollection. The findings demonstrate direct correspondence between brain activation correlates of recollection that have previously only been studied separately within fMRI and electrophysiological domains, thereby bridging research fields that have so far progressed in isolation.

## Figures and Tables

**Table 1:** Retrieval test accuracy and reaction times.

	Proportion correct		Reaction times (ms)	
	Mean	SEM	Mean	SEM
Location	0.84	0.02	1410	56
Task	0.80	0.02	1740	55
Occupation	0.80	0.02	1476	42

**Table 2:** fMRI activations associated with domain-general recollection.

Hemisphere	Region	BA	X	Y	Z	Voxels	Task > control T-value	Location > control T-value
*Task & location recollection* *>* *semantic control*								
Bilateral	Precuneus/superior parietal lobe/inferior parietal lobe	7/31/39/40	− 9	− 70	43	1536	9.19	10.28
Left	Middle/superior frontal gyrus	9/10	− 42	35	34	152	7.01	7.83
Bilateral	Cingulate gyrus	23	− 3	− 22	28	153	6.49	6.48
Right	Inferior parietal lobe	40	42	− 49	43	386	5.69	5.96
Right	Middle/superior frontal gyrus	10	33	56	10	148	5.35	5.81
Left	Middle frontal gyrus	6	− 33	5	52	75	5.21	5.26
Right	Middle frontal gyrus	6	39	8	52	34	4.63	5.7
Right	Middle frontal gyrus	9	42	32	31	32	3.97	4.09
Left	Superior frontal gyrus	10	− 24	56	− 2	19	3.79	3.79
Right	Insula	13	45	17	1	23	3.76	4.22

*Semantic control* *>* *task & location recollection*								
Bilateral	Anterior cingulate cortex/medial frontal gyrus	10/11/25/32	− 9	32	− 14	331	7.93	9.48
Left	Medial temporal lobe	28/35/36/Amy/Hip	− 24	− 16	− 17	124	6.35	8.07
Right	Medial temporal lobe	28/35/36/Amy/Hip	21	− 7	− 14	83	5.34	7.39
Right	Inferior frontal gyrus	47	36	32	− 14	10	4.78	7.65
Bilateral	Retrosplenial cortex	29/30	− 3	− 55	13	56	4.8	8.38

P < 0.001 uncorrected, > 10 voxels. Coordinates (x, y, and z) are cluster peaks from a conjunction analysis of the two simple effects in MNI space (Montreal Neurological Institute). T-values at these peaks are reported from simple contrasts. BA, approximate Brodmann area; Amy, Amygdala; Hip, Hippocampus.

**Table 3:** fMRI activations associated with domain-specific recollection.

Hemisphere	Region	BA	X	Y	Z	Voxels	T-value
*Task > location recollection*							
Left	Inferior frontal gyrus/sulcus/middle frontal gyrus	8/9/45/46/47	− 42	11	49	1130	8.2
Bilateral	Medial/superior frontal gyrus	6/8	− 3	17	52	345	5.62
Left	Inferior parietal lobe/supramarginal gyrus	39/40	− 48	− 52	43	343	4.99
Bilateral	Midbrain	Basal ganglia	− 9	− 22	− 14	53	4.85
Right	Middle frontal gyrus	8/9/45/46	54	29	28	93	4.8
Left	Middle temporal gyrus	21	− 48	− 34	− 2	70	4.58
Left	Lingual gyrus	19	− 27	− 76	− 8	39	4.35
Left	Precuneus	7	− 6	− 70	34	39	4.22
Bilateral	Retrosplenial cortex	29	− 3	− 43	22	56	4.17
Right	Supramarginal gyrus	40	60	− 55	37	17	4.05
Left	Diencephalon	Thalamus	− 12	− 13	10	17	3.8
Right	Posterior cingulate	30	18	− 70	10	14	3.8

*Location > task recollection*							
Right	Middle temporal gyrus	21	57	− 52	− 5	58	6.25
Right	Angular gyrus	39	45	− 76	28	24	4.94
Right	Postcentral gyrus	2/40	60	− 31	46	63	4.05
Left	Anterior cingulate cortex	24	− 6	23	− 5	13	3.72
Right	Superior frontal gyrus	8	24	11	49	11	3.7
Right	Precuneus	7	12	− 61	61	15	3.65

P < 0.001 uncorrected, > 10 voxels. Coordinates (x, y, and z) are in MNI space (Montreal Neurological Institute). BA, approximate Brodmann area.

## Footnotes

1 On additional location and task cued trials (36 in each condition), a “1” or “2” was displayed instead of a face picture in order to model cue-specific activations in the fMRI data (c.f. Simons, Gilbert, et al., 2005). However, there were no reliable activation differences between the cues and thus these conditions are not discussed further.

2 Because non-independent contrasts can bias conjunction p-values, common activation across both contrasts was defined as regions where each pairwise effect was independently significant at the specified threshold, and conjunction analysis was only used to localise the peaks in this overlap, with independent P-values reported for those peaks.

3 For the record, the only significant high frequency finding was a late onset, sustained (~ 1–2.5 s) event-related power suppression effect in the alpha-beta band (8–30 Hz) during Task recollection trials compared to the other conditions, which localised to sources in the temporal poles.

4 Although our effect had a more centro-parietal and bilateral distribution than the left parietal old/new effects seen in recognition tasks, this distribution is typical of tasks that require intentional contextual recollection (e.g. Allan and Rugg, 1997), and in particular, source memory judgements (e.g. Leynes, 2002; Wilding, 1999).
